# Pd–Co-Based Electrodes for Hydrogen Production by Water Splitting in Acidic Media

**DOI:** 10.3390/ma16020474

**Published:** 2023-01-04

**Authors:** Bernardo Patella, Claudio Zanca, Fabrizio Ganci, Sonia Carbone, Francesco Bonafede, Giuseppe Aiello, Rosario Miceli, Filippo Pellitteri, Philippe Mandin, Rosalinda Inguanta

**Affiliations:** 1 Dipartimento di Ingegneria, Università degli Studi di Palermo, 90128 Palermo, Italy; 2 Corpo Nazione dei Vigili del Fuoco, 41126 Rome, Italy; 3 IRDL UMR CNRS 6027, Université de Bretagne Sud, 56100 Lorient, France

**Keywords:** green hydrogen, electrolyzers, Pd–Co alloys, nanowires, template electrosynthesis, acidic media

## Abstract

To realize the benefits of a hydrogen economy, hydrogen must be produced cleanly, efficiently and affordably from renewable resources and, preferentially, close to the end-users. The goal is a sustainable cycle of hydrogen production and use: in the first stage of the cycle, hydrogen is produced from renewable resources and then used to feed a fuel cell. This cycle produces no pollution and no greenhouse gases. In this context, the development of electrolyzers producing high-purity hydrogen with a high efficiency and low cost is of great importance. Electrode materials play a fundamental role in influencing electrolyzer performances; consequently, in recent years considerable efforts have been made to obtain highly efficient and inexpensive catalyst materials. To reach both goals, we have developed electrodes based on Pd–Co alloys to be potentially used in the PEMEL electrolyzer. In fact, the Pd–Co alloy is a valid alternative to Pt for hydrogen evolution. The alloys were electrodeposited using two different types of support: carbon paper, to fabricate a porous structure, and anodic alumina membrane, to obtain regular arrays of nanowires. The goal was to obtain electrodes with very large active surface areas and a small amount of material. The research demonstrates that the electrochemical method is an ideal technique to obtain materials with good performances for the hydrogen evolution reaction. The Pd–Co alloy composition can be controlled by adjusting electrodeposition parameters (bath composition, current density and deposition time). The main results concerning the fabrication process and the characterization are presented and the performance in acid conditions is discussed.

## 1. Introduction

In recent years, the need to use fuels that are alternatives to conventional hydrocarbons has been increasingly growing. The indiscriminate use of fossil fuels is no longer sustainable, due to their limited availability and high environmental impact [1,2]. In the vast scenario concerning both fuels and energy conversion devices, electrochemical systems play a crucial role for hydrogen production and energy accumulation (primary and secondary cells and supercapacitors) as well as electricity production by means of fuel cells [3,4]. In particular, hydrogen obtained from the electrolysis of water is proposed as an alternative to fossil fuels in many applications [5,6]. There are several electrolyzer technologies (Alkaline [7,8,9], Proton Exchange Membrane (PEMEL) [10,11,12], anion exchange membrane (AEM) [13,14,15,16] and Solid Oxide Electrolyzer [17,18,19]) with a different degree of technological and commercial maturity. Generally, the energy used for electrochemical water splitting determines the “quality” of hydrogen and the associated environmental load [20,21]. In particular, the production of green hydrogen (i.e., hydrogen produced with energy from renewable sources) appears interesting, due to the absence of carbon dioxide emissions at all levels of the production chain [22,23], and it also allows the problems related to the unpredictable nature of renewable sources to be overcome [24,25]. In large electricity grids, renewable energy sources can be balanced by the production of energy from conventional sources, but the use of a higher percentage of renewable sources would require an accumulation system to balance energy variations when they are not directly consumed by the final user [26,27]. While batteries and capacitors are suitable for short-term electricity storage, long-term storage could be accomplished by employing hydrogen [28,29]. In such cases, the electricity produced from renewable sources would be used to feed the electrolyzers, thus being converted into hydrogen through the electrolysis of water. The hydrogen produced can be stored (for example in pressured tanks), and converted back into electricity when necessary, through fuel cells. In this way, the use of hydrogen as an energy vector allows the storage of excess energy during generation peaks from renewable sources and the production of energy in the phases of energy deficit. In this scenario, the production of hydrogen by means of PEMEL (polymer electrolyte membrane electrolysis) is particularly interesting because it adapts better to energy variability (particularly in the case of wind energy) with a faster response and better performance at a partial load [30,31]. There are still numerous limits to be overcome in coupling electrolyzers with renewable systems [32,33]. In particular, the lower and upper operating limits of the electrolyzer are important as these limit the exploitation of the energy produced by renewable systems and the number of stops permitted by manufacturers [34,35]. Further limits concern the electrolyzer, and, in particular, the cost of the materials used as electrocatalysts [36,37]. Notably, in the case of PEMEL, the most used electrocatalysts are those of the platinum family and, therefore, they are extremely expensive [38,39,40].

In considering of the potential of PEMEL, the main purpose of the present work was to study the fabrication procedure of a material with good electrocatalytic properties for the hydrogen evolution reaction (HER). In such regard, attention was paid to the preparation of Pd–Co alloys, which are characterized by their stability in the acid environment [41,42], due to their potentially good electrocatalytic properties, and their low cost compared to Pt [43], which is currently the material most frequently used for the electrochemical production of hydrogen. In particular, concerning the HER, Pd is characterized by a low hydrogen overvoltage; therefore, it is a solution candidate to replace Pt [36,44]. Additionally, electrodes made of Pd and Co alloys have been shown to be much more efficient catalysts than pure Pd or Co [45,46] and they are similar to those of Pt. As reported in literature, this is due to the synergistic effect of amorphous Co sites and Pd sites [47,48] present in the alloy. Carbon paper (CP) was chosen as the support for the electrocatalyst because it is commonly used in PEMEL for the manufacture of the membrane electrode assembly (MEA). CP is a material characterized by interconnected carbon fibers of micrometric dimensions, due to its structure and properties, and it has a high chemical stability, high surface area and good electrical conductivity. Furthermore, considering the excellent results obtained in the case of Ni-based nanostructured electrodes for alkaline electrolyzers [49,50,51], in this work, the Pd–Co alloys have been obtained in the form of an array of nanowires using alumina membranes (AAM) as the template [52,53,54,55]. In fact, as demonstrated in [56,57,58,59,60,61,62], due to the high surface area, the electrodes based on the arrays of nanowires showed very good performance compared to the planar ones. This is generally true for all electrochemical devices with nanostructured electrodes [4,63] such as batteries [64,65,66], sensors [67,68,69,70], supercapacitors [71,72,73] and solar cells [74,75,76].

For the fabrication of the electrodes, the electrochemical technique was chosen because it is flexible and allows the control of the composition of the alloy by tuning the electrodeposition bath composition [77,78]. As reported earlier, CP was chosen as a support for the electrodeposition processes and the deposition parameters were optimized to obtain a deposit that covered only the CP fibers. This guarantees a high surface area and, therefore, a good electrocatalytic performance. Moreover, it is essential that the deposition takes place only on the surface of the fibers and not on the entire area exposed to the solution, since this prevents the gas produced from escaping at the electrode/solution interface. Electrodes were tested for the HER at −50 mA cm^−2^ for 3 h. The electrochemical characterization was carried out in 2 M H_2_SO_4_ at 25 °C. For comparison, pure Pd and Co deposits were also prepared.

## 2. Materials and Methods

Commercial CP (Toray carbon paper, TGP-H-60) with a diameter of 1.5 cm (working diameter 1.3 cm) was used as support for the electrodeposition processes. For the fabrication of nanowires, AAM (the procedure for the fabrication of AAM is detailed in [53,79]) was used as a template, and its surface was sputtered before electrodeposition using a gold target to have a thin film of gold of approximately 300 nm that acted both as a current collector and as a mechanical support for the nanostructures.

The electrodeposition was conducted in a two-electrode cell, using a platinum mesh as a counter-electrode. The potentiostat used for electrodeposition was the P.A.R. Potentiostat/Galvanostat (mod. PARSTAT 2273, Princeton Applied Research, TN, USA). A tetrammine palladium dinitrate complex (Pd(NH_3_)_4_(NO_3_)_2_) was used for the electrodeposition process of palladium, while cobalt sulphate (CoSO_4_) was used for cobalt electrodeposition. For the deposition of the two pure metals, in both cases a solution of 0.035 M was used. All solutions were prepared with Millipore-grade water and analytical grade reagents. The cell, requiring only 0.5 mL of deposition solution, was specifically designed to reduce the consumption of expensive reagents, thus improving the overall cost-effectiveness of the process [80]. The depositions were carried out by imposing a constant current density optimized in the range between −1.56 and −156 mAcm^−2^ at 25 °C. Deposition times ranging from 1 min up to a maximum of 1 h were investigated.

In the case of Pd–Co alloys, solutions containing both Pd^2+^ and Co^2+^ at different ratios were used to study the effect of the bath composition on the deposit. Specifically, both the palladium-poor (containing Pd = 17.1% and Co = 82.9%) and the palladium-rich (containing Pd = 83.5% and Co = 16.5%) solutions were investigated. For each experiment, a fresh solution and an as-received CP was used. The loading of deposit on the CP support was evaluated by gravimetric measurements using a Sartorius microbalance (mod. Premium Microbalance ME36S, Sartorius, Germany).

The electrodeposition of NWs was carried out at a constant current density, −1.0 mAcm^−2^, for a time of 1 h at 25 °C using the same composition of the solutions reported above. After NWs deposition, the AAM was totally dissolved in 1.0 M NaOH at 25 °C.

The samples were characterized by X-ray diffraction (XRD, RIGAKU D-MAX 25600 HK, Tokyo, Japan), scanning electron microscopy (SEM, FEI QUANTA 200 FEG-ESEM, FEI, OR, USA), electron diffraction spectroscopy (EDS, EDAX, Ametek, PA, USA) and electrochemical analysis. EDS was performed in the full-frame mode (magnification 200 X, investigated area 2.25 mm^2^) in different areas of the sample to investigate its homogeneity. In the text, the average values of deposit composition are reported. In addition, EDS mapping was performed (magnification 2000x, investigated area 2.25 μm^2^). Also in this case, the analysis was carried out on different areas of the sample.

The electrochemical characterization was carried out by means of cyclovoltammetry (CV, scan rate of 5 mVs^−1^) and galvanostatic tests (at −50 mA cm^−2^ for 3 h). The current density referred to the geometric area of the electrode which is 2.24 cm^2^. For each sample, the CV was repeated for 100 cycles to evaluate the stability of the electrodes. The tests were carried out in H_2_SO_4_ 2 M, and as a reference electrode a mercury/mercury sulphate electrode (MSE) was used. All potential values reported in this work have been referred to the normal hydrogen electrode (NHE) at pH 0.

## 3. Results and Discussion

### 3.1. Deposit on Carbon Paper

To obtain cathode materials that can be used for HER in acidic aqueous solutions, binary alloys of Pd and Co have been synthesized on CP, that was used as a porous support for the gas diffusion layer (GDL). Table 1 (line 1) shows the SEM micrograph of the CP support before deposition. It is possible to observe that CP consists of interconnected carbon fibers determining a porous three-dimensional structure on a micrometric scale. EDS analysis shows the presence of a carbon peak (C), and a fluorine peak (F), the latter due to the polytetrafluoroethylene treatment of the CP to make it hydrophobic [81,82]. In the XRD patter the presence of a very intense peak at about 2θ = 26.7° is clear visible, which is characteristic of the crystallographic plane (002) of graphite [83,84]. Additionally, two minor peaks at 2θ = 53.5° and 2θ = 18° are present. The peak at 53.5° is related to the plane (004) of graphite, while the peak at 18° could not be identified and is probably due to impurities of the CP substrate.

Initially, the deposition process for pure Pd and pure Co was studied. This study is very important for determining the optimal conditions for the co-deposition of elements, whose standard reduction potentials have different values. In particular, the standard reduction potential for Co^2+^/Co is equal to −0.272 V/NHE, while for Pd^2+^/Pd it is equal to +0.9 V/NHE. The significant difference between the two standard reduction potentials complicates the co-deposition process, unless a chelating agent that limits the Pd activity is used [85]. For this reason, the tetrammine palladium dinitrate was used because the potential of ${\mathrm{Pd}(\mathrm{NH}}_{3}{)}_{4}^{2+}$/Pd is equal to 0.0 V/NHE due to the high stability of the complex [86,87].

The deposits obtained in the different conditions (at a different current density and deposition time) are characterized by a different quantity of electrodeposited material and by a different morphology. In particular, we found an increase of catalyst loading both with the increase of current density and deposition time. The morphology showed that for the samples obtained at a current density of −15.6 mAcm^−2^, the deposit has a needle-like structure as shown in Table 1 (line 2). This structure is present both for long and short deposition times. A needle-like structure, similarly obtained in [88], ensures a large active surface area and thus good electrocatalytic performance.

As the electrodeposition time increases, only the amount of deposit changes and, consequently, the coverage of the deposition area. Similarly, the samples obtained at −1.56 mAcm^−2^, as shown in Table 1 (line 3) show the previously described morphology. However, operating at a lower current density allows the reduction of the height of the Pd needles compared to those formed for the same times, but at −15.6 mAcm^−2^. A more detailed study of all the samples carried out on the internal CP fibers shows the presence of a globular morphology. This result indicates that the porous CP structure is not electrochemically uniform as demonstrated in [89,90]. The level of penetration of the electrolyte into the porous structure and, above all, the level of densification of the fibers at some points can determine the areas of higher or lower density of local current with consequent greater development of the needle-like structure, clearly visible in the SEM images reported in Table 1 (lines 2 and 3).

In the EDS spectra, the intensity of the Pd peak, that changes with the deposition time and with the deposition current density, indicates that the deposits are characterized by different quantities of electrodeposited material. The C and F peaks, also evident in the spectra, are both attributable to the CP substrate.

Regarding the crystallographic structure, the XRD patterns showed the presence of the diffraction peaks of the metallic Pd, which were identified by comparison with the database (card 87–637) [83,91]. In particular, the XRD peaks at 2θ = 40.0°, 46.8°, 68.1°, 82.2° and 85.5° indicate the presence of (111), (200), (220), (311) and (311) planes, respectively, and correspond to the face-centered cubic phase of Pd metal. The Pd deposit is polycrystalline with the most intense peak along the plane (111). Using the half-height width of the main peak and Scherrer’s formula [92], a grain size of about 15 ± 1 nm was calculated for the deposit obtained at −1.56 mAcm^−2^. This value changes very little with the deposition time, while it tends to increase slightly as the deposition current density increases, reaching a value of about 18 ± 1.3 nm in the case of the deposit obtained at −15.6 mAcm^−2^. The diffraction peaks of the CP substrate are also visible in the patterns. In the XRD patterns of the different samples only small differences were observed in terms of intensity of the Pd diffraction peaks, which were slightly lower in the samples obtained at the low deposition times and/or low current densities.

The electrodeposition process and the electrochemical behavior of pure Co was also investigated. The electrolytes for the electrodeposition of Co and its alloys are numerous, both acidic and alkaline, and usually include additives with various functions, from stabilizers, such as boric acid or ammonium citrate, to levelers and brighteners. The most widely used electrolytes are undoubtedly the acid solutions of sulphates, sulfamates, and, in specific cases, fluoborates and chlorides, both for the pure metal and for its alloys [86,93]. In this study, the Co electrodeposition process was carried out using a solution of cobalt sulphate 0.035 M. The electrodeposition was conducted at the same values of current density and deposition time employed for the Pd deposition. In the case of tests carried out at −15.6 mAcm^−2^, the Co deposit is present in all samples in increasing quantities as the deposition time increases. Instead, for the deposition carried out at −1.56 mAcm^−2^ it was observed that the Co deposit is present with evident quantities only in the samples obtained for times longer than 30 min.

The presence of the oxygen peak in the EDS spectra, especially at low current densities, suggests the probable co-deposition of Co oxides/hydroxides [94]. These compounds can be formed to the local alkalization of the solution due to the simultaneous evolution of hydrogen during deposition [95]. From a morphological point of view, it was found that all Co samples obtained at −15.6 mAcm^−2^ are characterized by a clod (dry mud) morphology that almost completely covers the CP surface. In fact, only a few fibers of the support remain uncovered as shown in Table 1 (line 4). This morphology is attributable to a dehydration effect that occurs during the air drying of the samples performed after deposition. The same morphology was also found at −1.56 mAcm^−2^, although the amount of deposit was much lower and only covered the surface of the fibers (Table 1, line 5).

For the crystallographic structure, regardless of the deposition conditions, the XRD results showed that all Co deposits are amorphous-nanocrystalline. Even if the spectra do not show perfectly evident peaks, through a smoothing operation it is possible to highlight the presence of some low intensity peaks. For comparison with the database (card 05–0727) [91], we have seen that the peaks at 2θ of 41.8°, 44.4° and 76.6°, indicate the presence of (100), (002) and (110) planes, respectively, and are attributable to the metallic Co with the hexagonal close-packed (hcp) structure [96,97,98]. The shape of the spectrum, in which the substrate peaks do not appear completely, the very low intensity of the Co peaks leads us to conclude that the deposit has a nanocrystalline near-amorphous nature.

After investigating the deposition process of pure metals, the electrodeposition process of the Pd–Co alloy was studied, to identify the optimal operating conditions (composition of the solution, current density and deposition time). The morphology showed that for depositions carried out at −15.6 mAcm^−2^ (Table 2, line 2) and −1.56 mAcm^−2^ (Table 2, line 1), the Pd–Co alloys have a globular structure only for very short deposition times (60 s). This structure changes significantly for longer deposition times. In fact, for a deposition time of 300 s, the deposits have a compact morphology formed by the coalescence of the globular structure. This effect is accentuated as the deposition time increases, with the tendency to form a compact deposit that tends to cover the entire surface of the CP. The samples were further characterized by EDS mapping. A uniform distribution of Co and Pd was observed in all deposition conditions, as evident in Table 2 (line 6), where a representative EDS map of Pd and Co was reported.

From the crystallographic point of view, for a content of Pd greater than 90%, and therefore for short deposition time, in the XRD patters the diffraction peaks of Pd are present, but they are very broad. Thus, the grain size is very small, and in fact, using Scherrer’s equation [92], an average size of 3.2 ± 0.3 nm was found. At 43.6° a very broad and low intensity peak is present which is assigned to (111) plane of cobalt palladium alloy with a cubic system (card 65–6174) [91,99]. The wideness and intensity of this peak suggest a very low crystallinity of the alloy also observed by other authors [100,101]. For long deposition times, due to the formation of Co rich alloys, the deposit becomes amorphous, due to the nature of Co. This same effect was also observed in other Co alloys obtained by electrodeposition [61,102,103].

For the samples obtained at −15.6 mAcm^−2^ (Table 2, line 4), due to the higher rate of the process and its intrinsic characteristic to enrich in cobalt, deposits with about 30% of Pd were obtained at 180 s, unlike the tests carried out at −1.56 mAcm^−2^ (Table 2, line 3) in which the percentage is still 65% for the same time. The morphology of these deposits is the globular type and the globular density increases with the deposition time and the deposition current density. For longer times, a compact deposit was obtained resulting from the coalescence of the globular structure. Conversely, for shorter deposition times it was possible to distinguish isolated grains. The nature of these deposits was not detectable by X-ray diffraction because they were amorphous or too thin, and in fact we obtained a spectrum similar to that of CP, which differed only by a lower intensity of the diffraction peaks.

Deposition from concentrated solution of Pd (about 84%) has led to the formation of very rich Pd deposit, as can be observed in Table 3 where the average composition calculated from the EDS results were reported. The Pd concentration decreases with increasing deposition time, remaining, however, above 56% for alloys obtained at −15.6 mAcm^−2^ and more than 40% for deposits obtained at −1.56 mAcm^−2^. For the deposition of the Pd–Co alloy from dilute solutions (about 17% in Pd), the formation of a Pd rich deposit is observed only for short deposition times. With the increase of deposition time the cobalt content increases (Table 4). This effect is attributable to the progressive Pd impoverishment in solution.

**Figure 1:**
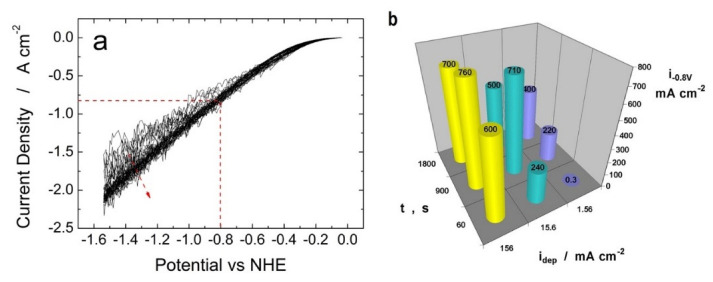
(**a**) The CV curves of the Pd–Co alloy obtained from dilute solution at −156 mA cm^−2^ for 900 s. (**b**) Current density values for HER (mAcm^−2^) at −0.8 V/NHE obtained from CV for the Pd–Co alloys obtained from a dilute solution (about 17% in Pd) at different deposition current density.

The electrochemical characterization of the deposits was carried out by means of voltammetry tests for 100 consecutive cycles, followed by a galvanostatic test carried out at −50 mAcm^−2^ for 3 h to verify the stability in acid environment under HER. All tests were carried out in 2 M H_2_SO_4_ at 25 °C. The CV was performed on an electrode area of 0.07 cm^2^, while for the galvanostatic test the entire electrode area, about 1.33 cm^2^, was used. Figure 1a shows the CVs for an alloy obtained at −156 mAcm^−2^ and for a deposition time of 900 s. For all investigated samples, the CVs have a similar trend to that shown in Figure 1a. Apart from the first cycles, the CV curves are settling and finally overlapping. At the same voltage, different current density values are reached, which were found to be functions of the deposition time and the deposition current density. To better compare the data obtained for the different samples, the current value of the HER at −0.8 V (NHE) was calculated from the CV. The results are shown in Table 5 for pure Pd and Co deposits and in Figure 1b for alloys obtained from the dilute Pd solution, (see also Table 3 for the results of the alloys obtained from the Pd-concentrated solutions). With the same procedure the as-obtained CP (without an electrocatalyst) was tested founding a hydrogen evolution current density of about −35 mAcm^−2^ at −0.8 V (NHE). This value was significantly lower than the current density values recorded at the same potential for the samples with the catalyst.

In the case of the pure Pd deposits, the hydrogen evolution current density at −0.8 V (NHE) increased as the deposition current density increased (Table 5). As predictable, a higher value of the deposition current density leads to a greater amount of deposit, and therefore, a greater electrocatalytic effect. In particular, a hydrogen evolution current density of −382 mAcm^−2^ was measured for the sample obtained for one hour of deposition at −1.56 mAcm^−2^, and a value of −412 mAcm^−2^ for the sample deposited at −15.6 mAcm^−2^ with the same deposition time. A similar behavior was observed for the sample of pure Co (Table 5). In fact, for the deposits obtained at −15.6 mAcm^−2^ and for a deposition time of 1 h, the current for hydrogen evolution was −281 mAcm^−2^ compared to −80 mAcm^−2^ measured for the deposits obtained at −1.56 mAcm^−2^ and with the same deposition time. As expected, the performance of the pure Co is worse than that of the pure Pd deposits [104].

In the case of the alloys, the best results were obtained from the diluted Pd solutions (Figure 1b). In fact, for the concentrated solutions, Table 3, hydrogen evolution currents were slightly superior to pure Pd (Table 5) were measured. Consequently, these alloys were not further investigated, since there was no improvement in their performance compared to the pure Pd. This result is attributable both to the low content of Co and to the small specific area. As discussed below, a high Co content is, instead, necessary to increase the electrocatalytic performance. The morphology of these deposits, especially for long deposition times, tends to become compact and thus have a low surface area. For alloys obtained from diluted solutions, the hydrogen evolution current changes greatly with the alloy composition, Figure 1b. Except for deposits obtained at −1.56 mAcm^−2^, the presence of a maximum deposition time of 900 s can be observed. Thus, this result is indicative that the addition of Co to Pd improves the electrocatalytic characteristics of the alloys up to an optimum value of approximately 90% after which, for further enrichment in Co, the benefits lapse. In particular, for alloys obtained at −15.6 mAcm^−2^, a hydrogen evolution current of −710 mAcm^−2^ is obtained at the maximum. This value is higher than −412 mAcm^−2^ that is the best value obtained for the pure Pd.

From the electrocatalytic point of view, considering these positive results of the deposits obtained from dilute Pd solutions, the investigation has been extended to deposits obtained initially from dilute solutions, but at a higher deposition current density of −156 mAcm^−2^. The morphology of these samples consisted in globular shapes that were coated with a visible patina, which however, was removed if the sample was treated in 2 M H_2_SO_4_ for a few minutes leaving a deposit with clear and well-defined contours (Table 2, line 5). An interesting aspect of these alloys is that the deposition affects not only the first layer of the CP fibers, but also the underlying fibers.

The electrochemical characterization of these deposits showed excellent electrocatalytic properties as shown by CV (Figure 1a). The hydrogen evolution current (Figure 1b) at −0.8 V/NHE increased with the deposition time for deposits up to 900 s, for which a value of −760 mAcm^−2^ was obtained. For deposits formed at longer times (1800 s) the hydrogen evolution current was −700 mAcm^−2^. At −156 mAcm^−2^, the maximum hydrogen evolution current was obtained, which was greater than that relative to the alloy obtained at −15.6 mAcm^−2^ (−710 mAcm^−2^); also higher than the value obtained for the pure Pd (−390 mAcm^−2^). Considering that the alloy composition obtained at −15.6 and −156 mAcm^−2^ was practically identical in both cases, this different behavior could be attributed to the morphological differences of the deposit, and in particular to a higher specific surface area obtained at −156 mAcm^−2^.

An exhaustive explanation of the presence of a maximum in the hydrogen evolution current corresponding to an alloy composition of the order of 10% in Pd is currently not available. The absence of a direct correlation between Pd content and the maximum in the hydrogen evolution current confirms the role of other parameters such as a deposited mass and its morphology. In the first approximation, it could be considered that for the deposition times that were shorter than the maximum, small amounts of deposit were obtained even if they were very rich in Pd. At longer times, however, the amount of deposit increased, as did the concentration of Co. Therefore, the presence of a maximum would be indicative of the best compromise between the quantity of deposit and its composition. The maximum performance for a specific quantity of Co present in the alloy was also found on other Co alloys [105]. Moreover, as reported by Li et al. [48], the presence of amorphous Co has a beneficial effect on the HER performance because it accelerates the water dissociation. However, the quantity of the deposit must also be considered in relation to its morphology, that is related to the specific surface area. In fact, as the deposition time increases, the deposit tends to become more compact; therefore, it has a smaller specific surface. It can be assumed, then, that the maximum corresponds to that optimal condition of the amount of deposit, the morphology and the content of Pd and Co.

**Figure 2:**
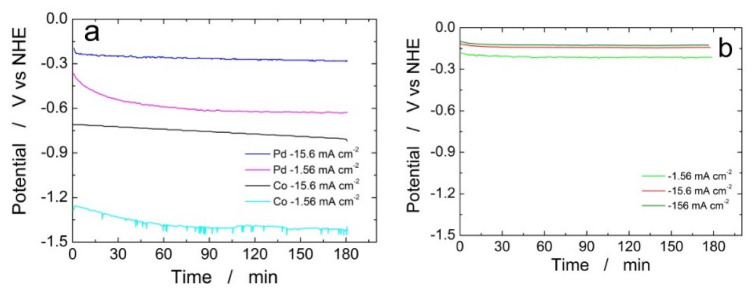
The galvanostatic test conducted at −50 mAcm^−2^ for 3 h in 2 M H_2_SO_4_ aqueous solution at 25 °C. (**a**) Pure Pd and Co deposits at different deposition current density for 3600 s. (**b**) Pd–Co alloys obtained from a dilute solution (about 17% in Pd) at different deposition current densities for 900 s.

Galvanostatic tests, Figure 2, showed that the HER from a 2 M H_2_SO_4_ solution at −50 mAcm^−2^ ran at different voltage values depending on the type of sample. In the case of the pure deposits (Figure 2a), it was noted that the electrode polarization was lower for deposits obtained at a higher deposition current density. This result can be explained in terms of the quantity of deposit present on the electrode. In other words, as the deposition current density increases, at the same deposition time, the deposit mass is higher so the electrode performance, from an electrocatalytic point of view, improves. The other observation is that the polarization of the electrode tends to increase progressively, at a rate that is greater for deposits obtained at a lower deposition current density. This can be attributed to the fact that during the HER there may be a gradual detachment of the deposit from the support resulting in a deterioration of the performance of the electrode. Obviously, this effect is more accentuated for deposits obtained at a lower current density since, as mentioned above, in this case its initial amount of deposit is lower. This effect is, however, of little relevance because a MEA-type assembly should significantly hinder this phenomenon.

For Pd–Co alloys a similar trend (Figure 2b) was observed. In fact, in the three hours of the test, the electrode polarization tended to decrease very slowly. It is interesting to note, however, that the electrode potential of the alloys was lower than the two pure metals, indicating better electrocatalytic properties. It was observed that for the deposit formed at −156 mAcm^−2^ an almost stationary value of the electrode voltage of −0.12 V/NHE was measured against the value of −0.27 V/NHE of the deposit of the pure Pd. This result confirms the best electrocatalytic behavior of the deposit formed at −156 mAcm^−2^, attributed to a higher specific surface area of this alloy and to the synergistic interactions between Pd and Co.

In Table 6 a comparison of our results with the reported performances in the literature relative to the Pd–Co-based electrodes is reported. This Table shows that our results align with the published data.

### 3.2. The Array of Nanowires

The fabrication procedure for nanowires of the Pd–Co alloy is detailed in [53]. The results showed that depending on the deposition conditions, it was possible to obtain arrays of both nanowires and nanotubes, and, as we have also shown in this work, it was possible to obtain both palladium-rich crystalline alloys and amorphous alloys rich in Co. In this study, to make a comparison with the data obtained using CP as a support for deposition, only arrays with about 8% of Pd were tested. In the case of deposits on CP, in fact, the alloys with this composition showed the best performances. According to the results discussed above, an array rich in Co is obtained when a dilute solution of palladium is used [53]. Also in this case, the alloy was found to be amorphous and the electrodes consisted of cylindrical nanowires (length around 4.5 μm), which replicated the morphology of pores of the alumina template.

**Figure 3:**
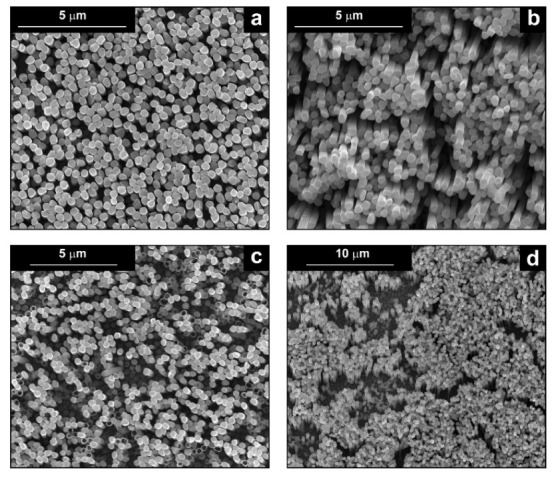
FESEM images of the Pd–Co alloy: (**a**,**b**) as prepared (**a**) top view, ((**b**) tilted top view), (**c**,**d**) after CV in 2 M H_2_SO_4_ (**c**) top view, ((**d**) tilted top view).

Before electrochemical characterization, to obtain an array of nanowires to be exposed to the H_2_SO_4_ solution, it is necessary to dissolve the alumina template in a NaOH solution. After this phase, the nanowires’ morphology is clearly visible, as shown in Figure 3a,b, which show that nanowires are uniformly distributed on the entire surface of electrode. However, the results obtained using this type of electrode were not satisfactory. Despite the first cycle of CVs having shown a very high hydrogen evolution current (about three times higher than the best result obtained for depleted alloys on CP), a rapid decay of the performance was observed. In fact, already after the 5th CV cycle, the hydrogen evolution current was lower than the value obtained in the case of CP. Analyzing the samples using the electron microscope showed that already after the first cycle of CV (Figure 3c,d) many nanowires had detached from the surface of the electrode. This was due to the vigorous hydrogen evolution, which makes the nanowires mechanically unstable. The loss of the nanowires leads to a decrease in the active area of the electrode and consequently to a drop in its performance.

To overcome this problem, the template used for the fabrication of arrays will be changed and a polycarbonate membrane will be used instead of the alumina membrane. A polycarbonate template is characterized by a lower porosity and consequently allows the fabrication of arrays with a lower density of nanowires, which have empty interspaces. This ensures a better evolution of the hydrogen from their surface and, therefore, a better mechanical stability. This hypothesis is confirmed by the good performance obtained with nanostructured Ni-based electrodes fabricated with polycarbonate membranes, that also have a very good mechanical stability [56].

## 4. Conclusions

In this paper, the study of the electrochemical deposition process of Pd–Co alloys on CP support and AAM was reported. The characteristics of the alloy were investigated through changing the compositions of the deposition bath, the deposition current density and the deposition time. SEM, EDS, XRD and electrochemical tests were performed in all samples to define the chemical–physical characteristics and the electrochemical performance. For comparison, pure Pd and Co deposits were also prepared in order to compare their performance with those of alloys. The results obtained show that the composition of the alloy is strongly dependent on all the parameters that control the electrodeposition process (time, current density and bath composition). In particular, the content of Pd decreases both with the increase of deposition time and the decrease of Pd concentration in the deposition solution. The best alloy was obtained at the high investigated deposition current density (−156 mAcm^−2^) and for 900 s of deposition time. Under these conditions, a Pd content in the alloy is of the order of 7–10%. The electrochemical characterization showed that, with this alloy a hydrogen evolution current at −0.8 V (NHE) of −760 mAcm^−2^ can be reached. The galvanostatic tests, performed for 3 h in 2 M H_2_SO_4_ and at 25 °C, also showed that in the case of this alloy the electrode potential remains roughly constant for the duration of the test. This result is an index of a good electrode stability.

Unexciting results were obtained in the case of the arrays of nanowires due to the lack of optimal mechanical stability of these structures under hydrogen evolution. New tests are in progress to overcome this problem, using polycarbonate membranes as templates for the fabrication of the nanowires.

## Figures and Tables

**Table 1:** The FESEM, EDS and XRD of the CP and Pd and Co deposits; the electrodeposition conditions and the composition of the electrodeposition bath is also reported.

Sample	FESEM	EDS	XRD
**Carbon Paper**	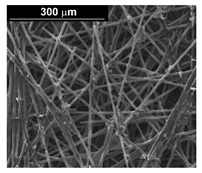	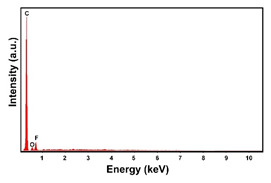	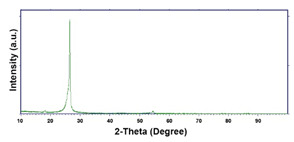
**Pd** (−15.6 mA cm^−2^, 3600 s)	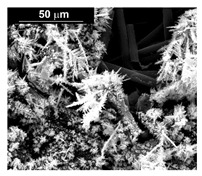	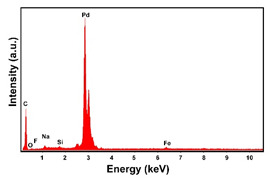	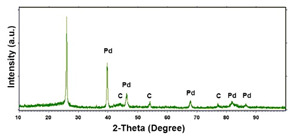
**Pd** (−1.56 mA cm^−2^, 3600 s)	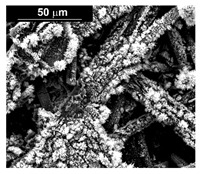	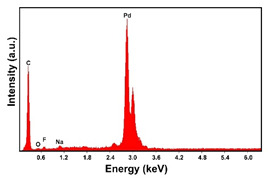	
**Co** (−15.6 mA cm^−2^, 3600 s)	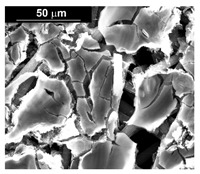	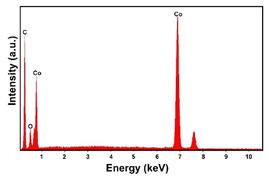	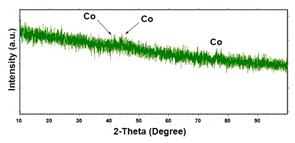
**Co** (−1.56 mA cm^−2^, 3600 s)	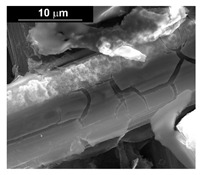	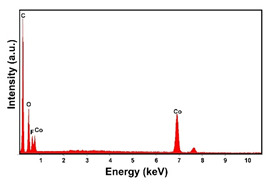	

**Table 2:** The FESEM, EDS and XRD of CP and Pd and Co co-deposits; the electrodeposition conditions and the composition of electrodeposition bath is also reported.

Sample	FESEM	EDS	XRD
**Pd–Co** (−1.56 mA cm^−2^ 0.035 M Pd^2+^, 0.0069 M Co^2+^, 60 s)	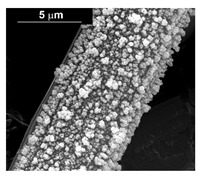	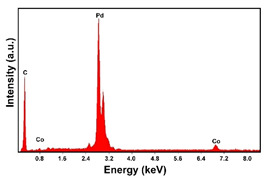	
**Pd–Co** (−15.6 mA cm^−2^ 0.035 M Pd^2+^, 0.0069 M Co^2+^, 300 s)	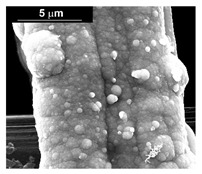	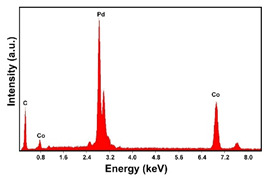	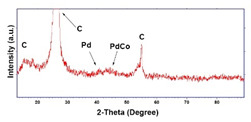
**Pd–Co** (−1.56 mA cm^−2^ 0.035 M Pd^2+^, 0.17 M Co^2+^, 60 s)	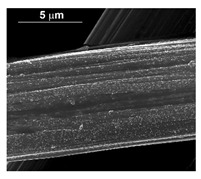	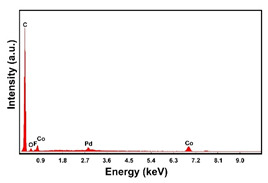	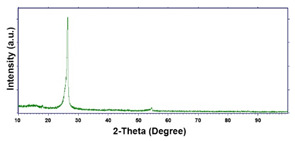
**Pd–Co** (−15.6 mA cm^−2^ 0.035 M Pd^2+^, 0.17 M Co^2+^, 60 s)	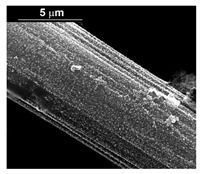	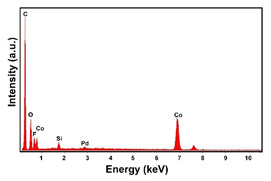	
**Pd–Co** (−156 mA cm^−2^ 0.035 M Pd^2+^, 0.17 M Co^2+^, 300 s)	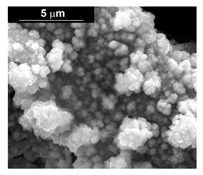	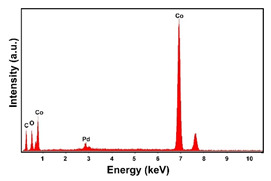	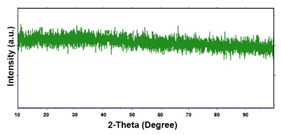
**Pd–Co** (−156 mA cm^−2^ 0.035 M Pd^2+^, 0.17 M Co^2+^, 300 s)	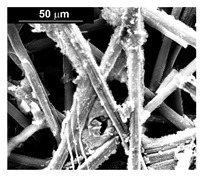	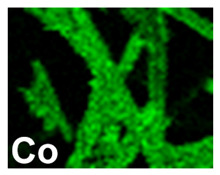	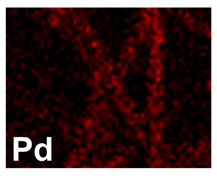

**Table 3:** The composition of Pd–Co alloys obtained from concentrate solution (about 84% in Pd) and current density values for HER (mAcm^−2^) at −0.8 V/NHE obtained from CV carried out in 2 M H_2_SO_4_.

Deposition Time, s	Pd–Co−1.56 mAcm^−2^		Pd–Co−15.6 mA cm^−2^	
	Pd%	i_−0_._8V(NHE)_ mAcm^−2^	Pd%	i_−0_._8V(NHE)_ mAcm^−2^
1800	40.6 ± 1.4	−242 ± 8	56.1 ± 1.2	−238 ± 10
900	65.8 ± 1.1	−233 ± 10	56.6 ± 1.6	−220 ± 12
180	75.7 ± 1.2	−391 ± 9	91.1 ± 1.4	−261 ± 7
60	80.6 ± 1.6	−415 ± 13	96.5 ± 1.2	−372 ± 8

**Table 4:** The composition of the Pd–Co alloy (Pd%) obtained from dilute solution (about 17% in Pd) at different deposition and current density.

Deposition Time, s	−1.56 mAcm^−2^	−15.6 mAcm^−2^	−156 mAcm^−2^
1800	16.9 ± 1.2	8.4 ± 1.1	5.1 ± 1.1
900	27.1 ± 1.1	9.8 ± 1.2	7.5 ± 1.2
180	65.2 ± 1.2	30.2 ± 1.2	47.9 ± 1.4
60	72.1 ± 1.1	45.5 ± 1.3	82.2 ± 1.2

**Table 5:** The current density values for HER (mAcm^−2^) at −0.8 V/NHE obtained from CV carried out in 2 M H_2_SO_4_ for samples of Pd and Co electrodeposited on carbon paper under different conditions.

Deposition Time, s	Pd		Co	
	−1.56 mAcm^−2^	−15.6 mAcm^−2^	−1.56 mAcm^−2^	−15.6 mAcm^−2^
3600	−382 ± 12	−412 ± 15	−80 ± 6	−281 ± 9
1800	−288 ± 11	−313 ± 12	−65 ± 4	−80 ± 6
900	−264 ± 10	−295 ± 10	−56 ± 4	−71 ± 4
60	−199 ± 6	−265 ± 9	−43 ± 4	−62 ± 6

**Table 6:** **A** comparison with the literature data.

Sample	Test Conditions	Potential for HER	Reference
Pd–Co@CN	0.5 M H_2_SO_4_ −36 mAcm^−2^	−0.1 V/NHE	[106]
Pd–Co alloy on carbon cloth	0.5 M H_2_SO_4_ −60 mAcm^−2^	−0.075 V/NHE	[107]
Pd–Co@N–C/rGO	0.5 M H_2_SO_4_ −10 mAcm^−2^	−0.158 V/NHE	[99]
Pd–Co on Carbon Paper	2 M H_2_SO_4_ −50 mAcm^−2^	−0.12 V/NHE	This Work

## Data Availability

Not applicable.
